# Neural Basis of the Emotional Conflict Processing in Major Depression: ERPs and Source Localization Analysis on the N450 and P300 Components

**DOI:** 10.3389/fnhum.2018.00214

**Published:** 2018-05-29

**Authors:** Jing Zhu, Jianxiu Li, Xiaowei Li, Juan Rao, Yanrong Hao, Zhijie Ding, Gangping Wang

**Affiliations:** ^1^Gansu Provincial Key Laboratory of Wearable Computing, School of Information Science and Engineering, Lanzhou University, Lanzhou, China; ^2^The Third People’s Hospital of Tianshui City, Tianshui, China

**Keywords:** major depressive disorder, emotional conflict, event-related potentials, source localization, standardized low-resolution brain electromagnetic tomography, N450, P300

## Abstract

**Objects:** Effective psychological function requires that cognition is not affected by task-irrelevant emotional stimuli in emotional conflict. Depression is mainly characterized as an emotional disorder. The object of this study is to reveal the behavioral and electrophysiological signature of emotional conflict processing in major depressive disorder (MDD) using event-related potentials (ERPs) and standardized low-resolution brain electromagnetic tomography (sLORETA) analysis.

**Method:** We used a face–word Stroop task involving emotional faces while recording EEG (electroencephalography) in 20 patients with MDD and 20 healthy controls (HCs). And then ERPs were extracted and the corresponding brain sources were reconstructed using sLORETA.

**Results:** Behaviorally, subjects with MDDs manifested significantly increased Stroop effect when examining the RT difference between happy incongruent trials and happy congruent trials, compared with HC subjects. ERP results exhibited that MDDs were characterized by the attenuated difference between P300 amplitude to sad congruent stimuli and sad incongruent stimuli, as electrophysiological evidence of impaired conflict processing in subjects with MDD. The sLORETA results showed that MDD patients had a higher current density in rostral anterior cingulate cortex (rostral ACC) within N450 time window in response to happy incongruent trials than happy congruent stimuli. Moreover, HC subjects had stronger activity in right inferior frontal gyrus (rIFG) region in response to incongruent stimuli than congruent stimuli, revealing successful inhibition of emotional distraction in HCs, which was absent in MDDs.

**Conclusion:** Our results indicated that rostral ACC was implicated in the processing of negative emotional distraction in MDDs, as well as impaired inhibition of task-irrelevant emotional stimuli, relative to HCs. This work furnishes novel behavioral and neurophysiological evidence that are closely related to emotional conflict among MDD patients.

## Introduction

Depression is among the most prevalent of all psychiatric disorders (First, 2013). Major depression disorder (MDD) has become a serious mental health issue, which always accompanies by severe symptom^1^. The cardinal features of depressive disorder are the impaired inhibition of task-irrelevant stimuli, especially to negative emotional information (Gotlib and Joormann, 2010). Typically, depressed individuals have difficulty in disengaging attention from negative thoughts, memories, and events in order to sustain attention toward on-going cognitive tasks (Siegle et al., 2002; Disner et al., 2011). In turn, susceptibility to emotional distraction adversely affects the ability of patients to respond to daily life needs. Activation abnormalities in brain areas implicated in emotional processing have been confirmed (Groenewold et al., 2013). However, the influence of negative emotions on depression in emotional conflict processing is still unclear.

Emotional Stroop tasks were commonly used to investigate the emotional conflict of depression in previous studies (Mcneely et al., 2008; Epp et al., 2012). In the research of Epp, subjects are instructed to identify the ink color of words that are either emotionally neutral (such as “apple”) or emotionally salient (such as “sad”), depressed subjects usually show longer reaction times (RTs) when naming the ink color of sad than neutral words in comparison to healthy controls (HCs) (Epp et al., 2012). However, the conventional emotional Stroop task does not directly reflect the conflict of emotional processing, since the emotional words and task-related information (ink color) are not in a lexical competition. Remarkably, report of face–word Stroop paradigm (Etkin et al., 2006) provides a feasible way to assess more directly the effects of emotional conflict in major depression. In this task, faces with happy and fearful expressions were presented with the words “happy” or “fear” shown above the facial expression, which yield emotional conflict. The face–word Stroop task is described in the previous literature and has been used in both healthy subjects and diseased subjects to investigate the emotional conflict (Etkin et al., 2006; Egner et al., 2008; Shen et al., 2013; Xue et al., 2017), but relatively few studies were found in major depression using this task.

Except for the researches of experimental paradigm, some researches of conflict control focus on the structural and functional aspects of brain. Here, we exploit some of the methodological insights gained from the study of cognitive conflict and apply them to emotional conflict. Literatures on cognitive conflict indicate that the anterior cingulate cortex (ACC) plays an important role in conflict monitoring (Botvinick et al., 2004; Holmes and Pizzagalli, 2008a) and response selection (Turken and Swick, 1999), similar findings have been reported in tasks involving conflict monitoring (Mansouri et al., 2009). Examination of a broad range of functional imaging, electrophysiological, as well as anatomical data in support of functional segregation has led some authors to divide the ACC into dorsal division for “cognitive” processes and ventral division for “affective” processes (Devinsky et al., 1995; Bush et al., 2000; Steele and Lawrie, 2004). The ventral division composed of rostral and subgenual components (Van Hoesen et al., 1993). Key regions such as the left rostral ACC and right precuneus have been proved to play a paramount role in emotion regulation of depression (Mitterschiffthaler et al., 2008). Furthermore, patients with frontal lobe injury that include the right inferior frontal gyrus (rIFG) are often impaired on inhibitory control tasks (Aron et al., 2003; Rubia et al., 2010). Dolcos and Mccarthy (2006) revealed a role of the IFG in inhibition of emotional distraction in healthy adults. Subjects with great activity in the IFG to emotional distracters tended to rate emotional distracters as less distracting, suggesting that activity in the IFG indexed successful inhibition of emotional distraction. Most of the above studies focused on the brain activation used the functional magnetic resonance imaging (fMRI) with high spatial resolution, but suffer from relatively low temporal resolution. On the other hand, event-related potential (ERP) has a higher temporal resolution, is inexpensive. Combination of high time resolution ERP and standardized low-resolution brain electromagnetic tomography (sLORETA) (Pascual-Marqui, 2002) can effectively solve the inverse source imaging.

The ERP is an impactful technique to explore brain activities (Shen et al., 2013; Zhao et al., 2017a) and has been used to investigate conflict control. So far, researchers observed that two ERP components were related to conflict processing: N450 and P300. N450 component is defined as a negative voltage deflection peaking approximately 450 milliseconds after stimulus presentation with origins in a frontocentral scalp distribution. The N450 component is often assumed to be an index of conflict detection and conflict monitoring, most likely in the response stage (West, 2003) and showed greater N450 amplitude following incongruent stimuli than following congruent stimuli. The P300 is a positive-going potential at parietal electrode sites which peaks around 300–600 ms window following the stimulus onset (Zhao et al., 2017b). P300 component reflects the conflict resolution process because the component is considered to index the stimulus assessment and reflect the sensitivity to the response selection process (Duncan, 1981; Xue et al., 2017). MDDs exhibited abnormal conflict processing in cognitive conflict tasks. As an example, Holmes and Pizzagalli (2008b) found a greater Stroop effect on the RT in depressive patients, and the Stroop effect for N450 component was absent in patients with depression relative to healthy subjects. Another research reported that both the overall N450 amplitude and N450 congruent effect were attenuated in low-performing depression (Holmes and Pizzagalli, 2008a). Moreover, a study of patients with MDD by Dai and Feng (2011) found a smaller P300 amplitude at parietal sites relative to HC subjects using emotional Stroop paradigm. These findings supported that P300 and N450 are all involved with conflict processing. Combined with ERP source imaging, we were interested in exploring the neural mechanisms underlying the emotional conflict processing in MDDs during a face–word Stroop task.

In the current study, we used a face–word Stroop task to examine how the MDD influences the neural process during emotional conflict. In this task, we used emotional faces as our experimental materials. The components of P300 and N450 associated with conflict processing were extracted for further analysis. On the behavioral level, compared with HCs, we hypothesized that MDD patients would have increased RT Stroop effect in response to happy incongruent stimuli with negative distraction word and happy congruent stimuli with positive distraction. In addition, on the neurological level, we expected MDD patients would demonstrate certain abnormal neurophysiological indicators, which might be manifested as (i) attenuated difference between P300 component to sad congruent stimuli and sad incongruent stimuli; (ii) enhanced rostral ACC activity to happy incongruent stimuli compared with happy congruent stimuli. Further, we hypothesized the rIFG would be recruited under successful suppression of interference in HCs.

## Materials and Methods

### Participants

Twenty patients with MDD (aged 32.65 ± 9.6 years; 11 females and 10 males; education level 10.70 ± 3.40 years) and 20 HCs (aged 30.85 ± 9.82 years; 10 females and 10 males; education level 12.10 ± 2.35 years) participated in the experiment. All participants were right-handed, with normal or corrected-to-normal visual acuity. The diagnosis of MDD was determined by a psychiatrist using the Mini International Neuropsychiatric Interview (MINI) (Lecrubier et al., 1997), which was based on the Diagnostic and Statistical Manual of Mental Disorders-IV (DMS-IV) (O’Shea, 1989) and the International Classification of Diseases-10. The HC individuals were free of brain illness history, psychiatric disorders, or medications. Meanwhile, the Patient Health Questionnaire-9 (PHQ-9) was used to assess the severity of depressive symptoms in both groups. Independent *t*-tests were performed to test the difference between two groups, in terms of the age, education, and PHQ-9 scores, respectively, while group difference in the gender was examined by using the Chi-squared test. There are no significant differences between MDD patients and HCs in age (*t*_38_ = -0.95, *p* = 0.56), gender (χ^2^ = 0.75, *p* = 0.50), and education level (MDD, 10.70 ± 3.40 years; HC, 12.10 ± 2.35 years; *t*_38_ = 1.51, *p* = 0.15). The PHQ-9 scores from MDD patients were significantly higher than those from HCs (18.75 ± 4.34 for MDD patients; 0.62 ± 1.90 for HC individuals; *t*_38_ = 17.09, *p* < 0.0005). Moreover, the comorbidity anxiety was assessed by psychiatrists using the MINI and the Generalized Anxiety Disorder Scale-7 (GAD-7). The depressive participants with high generalized anxiety symptom were excluded. All participants provided written informed consent before enrolment in the study, which was approved by The Third People’s Hospital of Tianshui City’s ethics committee. Each subject would receive 100 China Yuan for the participation after experiment.

### Design and Implementation of the Face–Word Stroop Task

**FIGURE 1:**
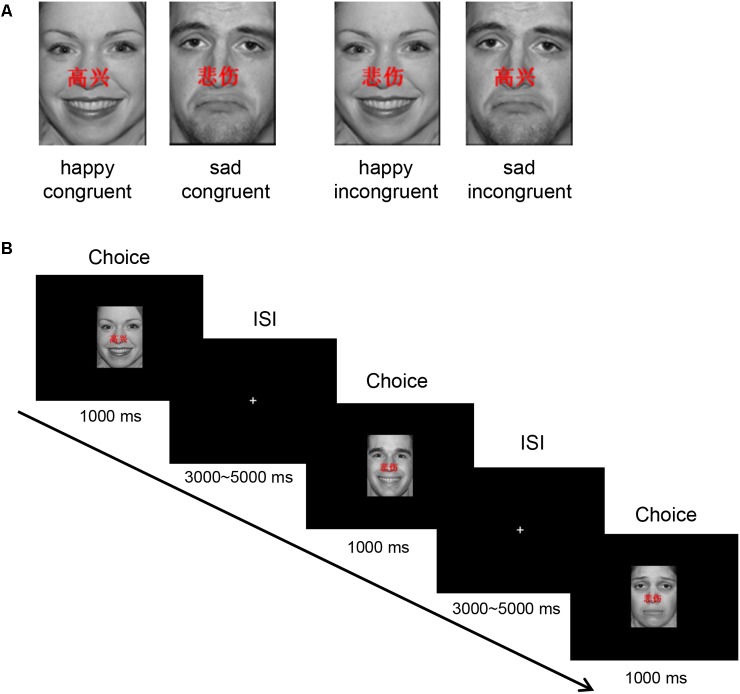
Experimental protocol. **(A)** An example of stimuli materials for each condition. **(B)** Example timelines used in the face–word Stroop task. Participants needed to complete the identification of facial affect with happy or sad expressions that had either “
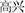
” (which means “happy”) or “
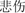
” (which means “sad”) word written across them.

Forty happy and 40 sad faces were selected from the international affective pictures systems (IAPSs) (Lang et al., 1997) for use during this study. The faces measured 10.84 cm × 8.13 cm, 240 pixels/inch, and all non-facial features were trimmed (i.e., no hair or clothing). We used Matlab to equate mean pixel luminance, contrast, center-spatial frequency of all faces. The happy facial expression with “
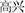
” and sad facial expression with “
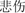
” were defined as happy congruent condition and sad congruent condition, respectively, whereas the happy facial expression with “
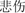
” and sad facial expression with “
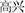
” were defined as happy incongruent and sad incongruent conditions, respectively. An example of stimuli materials for each condition is shown in **Figure 1A**. Gender and facial expression were balanced across responses and trial type.

The experimental protocol is shown in **Figure 1B**. In each trial, stimulus is displayed for 1000 ms in the center of the black screen, and then a central-fixation cross is presented with a varying inter-stimulus interval (ISI) for 3000–5000 ms (mean ISI = 4000 ms). Participants are instructed to press keys as quickly and accurately as possible to identify the emotion of the presented facial expression, while ignoring emotionally congruent or incongruent words, by pressing button “left” and “right” corresponding to the happy (right middle finger) and sad (right index finger) facial expressions, respectively.

The task consisted of 160 trials of happy or sad facial expressions with the red letters “
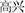
” (which means “happy”) or “
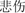
” (which means “sad”) shown above the facial expression. The number of trials for each condition is equal (40 trials), with a pseudo-random order. IBM compatible computers and DELL 17-I were used to present this task which was programmed with E-Prime version 2.0 software (Psychology Software Tools Inc., Pittsburgh, PA, United States). Participants were asked to complete four practice trials and reach an accuracy rate (AR) of over 75% before the real experiment. The experiment took place in sound attenuation, light dark down, and air conditioning room.

### EEG Data Acquisition and Preprocessing

EEG was recorded by Net Station software (version 4.5.4) using a 128-channel HydroCel Geodesic Sensor Net site. Impedance of each electrode was kept below 60 kΩ. The sampling rate was 250 Hz. The Cz electrode then EEG data were re-referenced offline to average reference (Liu et al., 2015). EEGLAB (Delorme and Makeig, 2004) toolbox and ERPLAB (Lopezcalderon and Luck, 2014) toolbox in Matlab were used to process and analyze the continuous EEG data. Specifically, the EEG data were filtered by high-pass filter with a cutoff frequency of 0.1 Hz and low-pass filter with a cutoff frequency of 30 Hz. We removed the section of EEG data with large muscle noise or extreme voltage offset by visual inspection, such as swallowing and coughing. Components that associated with eye movements and eye blinking activities were identified and removed by performing independent component analysis (ICA) for each participant (Jung et al., 2000).

### Analysis of Behavioral Data

For behavioral data, we mainly focused on analyzing behavioral adjustments related to the occurrence of conflict responses. According to previous studies (Holmes and Pizzagalli, 2008b; Epp et al., 2012; Schmidt, 2013), we calculated the Stroop effect and Gratton effect. The Stroop effect is a measurement of interference caused by incongruent stimuli compared with congruent stimuli. It is calculated as: (RT_incongruent trials_ - RT_congruent trials_) and (Accuracy_congruent trials_ - Accuracy_incongruent trials_). As a result, the larger scores are indicative of increased Stroop interference effect (Holmes and Pizzagalli, 2008b). The Gratton effect is a demonstration of post-conflict behavior adjustment. It is calculated as: (RT_incongruent trials - following congruent trial_ - RT_incongruent trials following incongruent trial_) and (Accuracy_incongruent trials following incongruent trial_ - Accuracy_incongruent trials following congruent trial_). The higher scores are indicative of increased cognitive control (Epp et al., 2012). For Gratton effect, we only examined the trials with the correct response for analysis of post-conflict adjustment, in order to distinguish the post-conflict and post-error adjustment effects (Pizzagalli et al., 2006). The number of trials with high conflict trials and low conflict trials in each group was about 40. Numbers of trials remaining for each trial type were balanced.

### Analysis of ERP Components

In order to measure the ERP waveforms, ICA-corrected EEG data were segmented into epochs starting at 200 ms prior to stimuli onset until 1000 ms after the stimuli appeared, and baseline corrected using -200 to -100 ms pre-stimuli. Approximately 7% of all trails (the signal exceeded ±100 μV) for each emotional face in congruent and incongruent conditions were excluded. Statistical results manifested that numbers of trials remaining for each trial type had no significant difference (*p* > 0.05).

Event-related potential analyses mainly focused on components of N450 and P300. Based on the previously reported literature (Krompinger and Simons, 2011; Xue et al., 2017) and scalp topographies, in the present study, the mean amplitude and latency of the N450 were measured between 350 and 700 ms following the stimuli onset at average electrodes of AF3, AF4, FP1, FP2, F5, and F6. The P300 was defined at average electrodes of CPz and Pz with a time window between 300 and 600 ms after stimuli presentation.

### Standardized Low-Resolution Brain Electromagnetic Tomography

To identify neural generators involved in emotional conflict of major depression and their corresponding functional roles in the emotional conflict control, the brain sources of each ERP component were reconstructed using sLORETA (Pascual-Marqui, 2002). The sLORETA is a functional source imaging method based on a three-shell spherical model registered to the Talairach human brain atlas, available as a digitized MRI from provided the Brain Imaging Centre, Montreal Neurological Institute (MNI, Talairach and Tournoux, 1988). The spatial extent of the current density includes cortical gray matter and hippocampal area, a total of 6239 voxels at 5 mm spatial resolution. At each voxel, the dipole moment of three directions was estimated. The sLORETA uses the minimum norm criterion to estimate the current density, positioning according to the local maximum current density. According to our previous research (Li et al., 2015), 69 scalp electrodes were used in source localization analysis to answer two questions, i.e., “When” and “where.” The “When” question tries to position the difference temporally and the “Where” question tries to position these differences in the three-dimensional space within the brain.

In order to answer the “When” problem, paired and independent *t*-tests were calculated for all 300 time-samples of each epoch (4 ms each per sample, i.e., 1200 ms) to examine the differences between conditions and between groups, respectively. For each comparison, we selected separately about 10 significant time-samples (*p* < 0.05, two-tailed) within the time ranges of P300 and N450 components for further analysis. The significance level was corrected for multiple comparisons and false positives (Nichols and Holmes, 2002). Afterward, in order to answer the “Where” question, sLORETA values were created from these time-samples for each condition and group. Paired and independent *t*-tests were calculated to identify the differences between the conditions and between the groups. For each comparison, source localization results of ERP data of each trial type were compared voxel-by-voxel using an independent log-F-ratio statistic test, and found significant activation of brain areas at *p* < 0.05.

### Statistical Analyses

#### Behavioral Data

Only trials in which participants made responses were considered. We also excluded the trials with RT that exceeded the individual mean ± 2 standard deviation (SD) for each trial type.

For the Stroop effect, mixed 2 × 2 ANOVA with group (MDDs vs. HCs) as a between-subjects factor and face_score_ (Stroop scores for emotional faces, happy_stroop_ vs. sad_stroop_) as a within-subject factor was conducted on RT and AR. For the Gratton effect of RT and AR, independent *t*-tests were performed to test the difference between MDDs and HCs. As described above, merely the trials after the correct trial were considered.

#### ERP Data

To evaluate the characteristics of emotional conflict in MDDs, we compared ERP components between MDDs and HCs. In particular, the analyses mainly focused on N450 component associated with conflict monitoring and P300 associated with conflict resolution processing. Repeated-measure ANOVAs with face (happy vs. sad) and congruency (congruent vs. incongruent) as within-subjects factors and group (MDDs vs. HCs) as between-subjects factor were conducted for the mean amplitude and latency of P300 and N450, respectively. Greenhouse–Geisser corrections were applied. In these analyses, we corrected for multiple comparisons with the Bonferroni method.

## Results

### Behavioral Measures

The average RT and AR scores of two groups for the Stroop effect and the Gratton effect are described in **Table 1**.

**Table 1:** Summary of behavioral, the ERP average of channels AF3, AF4, FP1, FP2, F5, and F6 for the N450 component and the ERP average of channels CPz and Pz for the P300 component in the MDDs and HCs.

		MDDs	HCs
*A. Behavioral performance*	
Stroop scores RT	Happy face	27.42 (18.87)	10.36 (24.58)
	Sad faces	11.67 (24.29)	15.52 (26.63)
Stroop scores AR	Happy face	5.00 (9.17)	5.62 (6.00)
	Sad faces	0.37 (5.63)	2.37 (6.04)
Gratton scores RT		6.4 (26.56)	14.61 (27.19)
Gratton scores AR		3.4 (7.97)	2.93 (5.24)
*B. Scalp ERP data*	
N450 amplitude (μV)	Happy congruent	-0.70 (4.25)	-0.4861 (9.76)
	Happy incongruent	-1.25 (6.29)	-1.03 (9.41)
	Sad congruent	-1.05 (4.92)	-0.99 (7.27)
	Sad incongruent	-1.65 (4.89)	-1.57 (9.42)
N450 latency (ms)	Happy congruent	620.07 (78.19)	487.27 (42.52)
	Happy incongruent	590.80 (59.81)	498.37 (44.62)
	Sad congruent	621.13 (76.61)	596.467 (88.62)
	Sad incongruent	615.37 (95.69)	489.07 (54.33)
P300 amplitude (μV)	Happy congruent	4.51 (2.72)	3.10 (3.96)
	Happy incongruent	4.34 (3.23)	2.63 (3.60)
	Sad congruent	4.47 (2.78)	2.00 (3.42)
	Sad incongruent	4.78 (3.25)	3.11 (3.65)
P300 latency (ms)	Happy congruent	497.60 (56.53)	514.40 (53.53)
	Happy incongruent	502.00 (45.84)	504.60 (66.63)
	Sad congruent	494.50 (58.48)	471.80 (58.80)
	Sad incongruent	498.10 (54.69)	490.90 (53.31)


#### Stroop Effect

Statistical analysis on the RT did reveal a significant interaction effect of face_score_ × group (*F*_1,38_ = 4.06, *p* = 0.05). No other significant effects emerged. To further explore this interaction of between face_score_ and group, *post hoc* Newman-Keuls tests were performed. Results revealed that the difference between groups was significant in responding to happy facial expression (*F*_1,38_ = 6.12, *p* < 0.02), indicating that the Stroop score between happy incongruent trials and happy congruent trials in MDDs was significantly higher than those of HC group.

ARs revealed a significant main effect of face_score_ (*F*_1,38_ = 6.00, *p* < 0.02), with higher Stroop scores to identify happy facial expression than to identify sad facial expression in participants. No other main effects or interactions obtained significance.

#### Conflict-Adaptation (Gratton) Effects

For both RT and AR, independent *t*-tests result showed that there are no significant differences between MDD patients and HCs.

### ERP Waveforms of Two Groups in Face–Word Stroop Task

**FIGURE 2:**
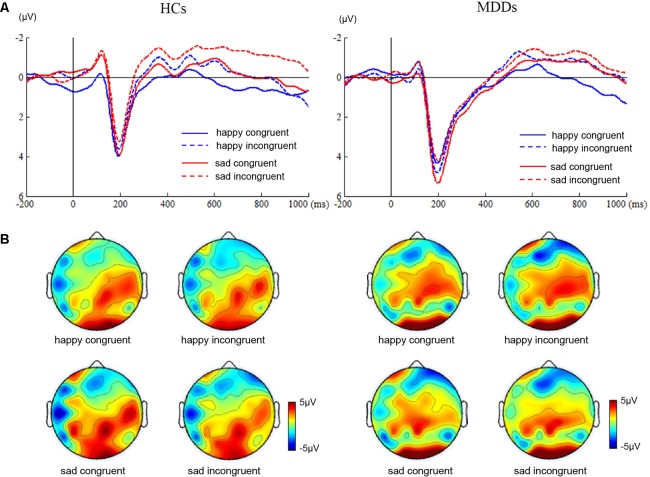
ERP waveforms and scalp topographies of N450. The waveforms were averaged across AF3, AF4, FP1, FP2, F5, and F6 to reflect the N450 ANOVA findings **(A)**. The corresponding scalp topographies elicited by the target congruent stimuli and incongruent stimuli were obtained for a 20-ms long time interval around the latencies of the N450 peaks **(B)**.

For amplitude of N450, no significant main or interaction effect was found. For latency of N450, there was a significant difference between two groups (**Figure 2**, *F*_1,38_ = 28.72, *p* < 0.0005). Furthermore, the results of statistical analysis revealed significant main effects of face (*F*_1,38_ = 9.94, *p* < 0.01) and congruency (*F*_1,38_ = 14.14, *p* < 0.01), and a two-way interaction of face × congruency (*F*_1,38_ = 8.54, *p* < 0.01, and a three-way interaction of face × congruency × group (*F*_1,38_ = 19.07, *p* < 0.0005) were significant. To further examine the three-way interaction, a simple effect test was performed. It showed a significant effect of congruency for happy facial expression in MDD group (*F*_1,38_ = 5.30, *p* < 0.02), and a significant effect of congruency for sad facial expression in HC group (*F*_1,38_ = 28.13, *p* < 0.0005). The HC individuals manifested shorter N450 latency at sad incongruent trials than at sad congruent trials. However, MDD group had shorter N450 latency at happy incongruent trials than at happy congruent trials.

**FIGURE 3:**
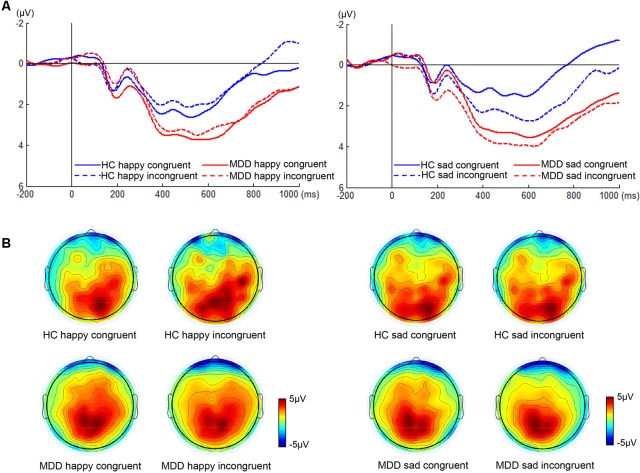
ERP waveforms and scalp topographies of P300. The waveforms were averaged across electrodes CPz and Pz to reflect the P300 ANOVA findings **(A)**. The corresponding scalp topographies elicited by the target congruent stimuli and incongruent stimuli were obtained for a 20-ms long time interval around the latencies of the P300 peaks **(B)**.

We found significant differences in P300 amplitude between two groups (**Figure 3**, *F*_1,38_ = 4.75, *p* < 0.05). A significant interaction of face × congruency (*F*_1,38_ = 6.23, *p* < 0.02) and a three-way marginal interaction of face × congruency × group (*F*_1,38_ = 4.04, *p* = 0.052) were observed. No significant main or interaction effect was found. To further examine the three-way marginal interaction, a simple effect test was performed. It showed a significant effect of congruency for sad facial expression in HC group (*F*_1,38_ = 6.96, *p* < 0.02), such that P300 latency following sad incongruent stimuli was significantly higher than sad congruent stimuli in HCs. For P300 latency, no significant main or interactions effects were found. The detailed amplitude and latency of P300 and N450 were summarized in **Table 1**.

### sLORETA: Between Groups and Conditions Differences for ERP Components

**FIGURE 4:**
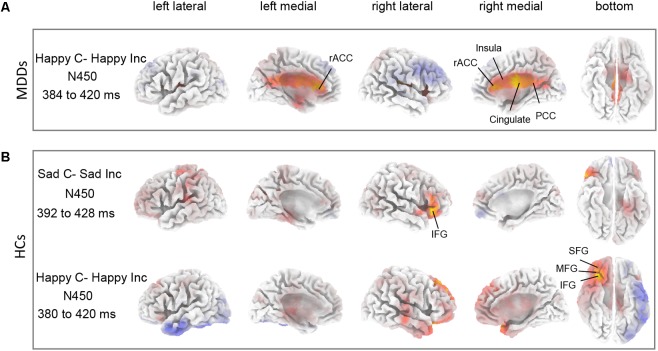
Differences between task conditions. The statistical differences between congruent and incongruent task conditions are shown for HC **(A)** and for MDD **(B)** groups within N450 time window. Yellow color indicates significantly higher estimated current density of incongruent trials compared with congruent trials. The time periods for different components are also shown in **Table 2**.

The sLORETA statistical non-parametric maps from within-subject comparison and between-subject comparison within N450 and P300 time windows were shown in **Figure 4**. Note that these maps represent log-F-ratio statistic results for each comparison. HC group exhibited a significant difference between current densities of congruent trials and incongruent trials with activation during incongruent trials being higher (**Figure 4**). As illustrated in **Table 2**, the differences revealed significantly increased inferior frontal gyrus [IFG: Brodmann area (BA) 47] activation to happy incongruent stimuli than happy congruent stimuli, as well as enhanced IFG, superior or medial frontal gyrus (SFG/MFG: BA 6/8/9) activation to sad incongruent stimuli relative to sad congruent stimuli at the N450 time window. However, MDD patients demonstrated a pattern of significantly increased current density in rostral ACC (BA 24/32/33), posterior cingulate cortex (PCC: BA 23/30/31), cingulate gyrus (BA 23/24), and insula (BA13) activation to the happy incongruent stimuli than happy congruent stimuli at N450 time window.

**Table 2:** Comparison of congruent and incongruent tasks as well as HCs and MDD individuals in the time ranges of P300 and N450 components in sLORETA.

Lobe	Structure	OBA	Activity	MNI
*A. Differences between task conditions in HCs*	

(a) N450 sad congruent trials < sad incongruent trials (392–428 ms)				

Frontal lobe	Inferior frontal gyrus	47R	↑	30, 30, -20
Frontal lobe	Middle frontal gyrus	11R	↑	30, 35, -20
Frontal lobe	Superior frontal gyrus	9R	↑	20, 50, 40

(b) N450 happy congruent trials < happy incongruent trials (380–420 ms)				

Frontal lobe	Inferior frontal gyrus	47R	↑	45, 30, -5

*B. Differences between task conditions in MDDs*	

(a) N450 happy congruent trials < happy incongruent trials (384–420 ms)				

Sub-lobar	Insula	13R	↑	35, -20, 20
Limbic lobe	Cingulate gyrus	23/24R	↑	5, -10, 30
Limbic lobe	Cingulate gyrus	24/31L	↑	-5, -5, 30
Limbic lobe	Anterior cingulate	24/33L	↑	-5, 25, 15
Limbic lobe	Anterior cingulate	24/32/33R	↑	5, 30, 15
Sub-lobar	Insula	13L	↑	-35, -15, 20
Limbic lobe	Posterior cingulate	23R	↑	5, -30, 25
Limbic lobe	Posterior cingulate	23L	↑	-5, -45, 25
Limbic lobe	Posterior cingulate	23/30M	↑	0, -50, 20

*C. Differences between groups*	

(a) N450 for happy incongruent trials (360–400 ms) MDDs < HCs				

Frontal lobe	Precentral gyrus	6R	↓	45, -5, 55

(b) P300 for sad incongruent trials (480–520 ms) MDDs > HCs				

Parietal lobe	Inferior parietal lobule	40R	↑	40, -55, 50
Parietal lobe	Superior parietal lobule	7R	↑	40, -60, 50


*OBA, orientation Brodmann area; R, right; L, left; M, medial; ↑, significantly increased neural activity; ↓, significantly decreased neural activity; MNI, Montreal Neurological Institute.*

Groups differed was significant solely on the current density of incongruent stimuli. As we demonstrated in **Supplementary Figure S1**, for happy incongruent trials, sLORETA analysis suggested that MDD patients had lower activity in precentral gyrus (BA 6) within the N450 time window relative to HC individuals. For sad incongruent trials, a higher estimated current density in precuneus (BA 7/19), as well as superior and inferior parietal lobule (SPL/IPL: BA 7/40) were observed in MDD patients for P300 component compared with HCs. The difference of congruent conditions was not significant between groups. The summary of the results is presented in **Table 2**.

### Correlations

To examine the relationship between behavior and electrophysiological signature, we correlated the RT Stroop score and AR Stroop score separately with the difference in amplitudes of the P300 and N450 on emotion-incongruent and emotion-congruent trials. Pearson’s correlation test was used to assess the correlation between variables. A negative relationship of RT Stroop score with the difference in P300 amplitude between sad incongruent and sad congruent trials was observed in HCs (*p* = 0.002, *r* = -0.64). This negative correlation was lost in MDDs. The difference in P300 amplitude as a function of each of the RT measure is shown in **Supplementary Figure S2**.

## Discussion

The goal of present study was to examine the impact of sad and happy emotions on major depression in response to emotional deficit, with a face–word Stroop task in which emotional faces as experimental materials. The following findings emerged. Firstly, for Stroop effect, MDD subjects were characterized by significantly increased scores between RT of happy congruent trials and RT of happy incongruent trials, relative to HC subjects. Since the Stroop effect is a measurement of interference elicited by the incongruent trials, relative to congruent (Holmes and Pizzagalli, 2008b; Schmidt, 2013; Braverman and Meiran, 2015), higher Stroop scores indicated increased interference effects. This finding manifested that MDDs had increased RT interference effects to happy incongruent trials in comparison to HCs. For both sad congruent trials and sad incongruent trials, no group differences for Stroop scores emerged in our study. The models of Beck et al. (1979) and Bower (1981) predict that depression associated with an attentional bias for mood-congruent stimuli. This result suggests that the emotional conflict processing in major depression was characterized by an attentional bias for negative distractive stimuli and need enhanced cognitive control. Moreover, participants showed higher Stroop scores to identify happy facial expression than to identify sad facial expression in AR, indicating that happy congruent trials than sad incongruent trials induced enhanced interference effects. However, for Gratton effect, comparison of post-conflict behavior adjustment in two groups had no significant difference in RT and AR.

Secondly, patients with MDD failed to show obvious Stroop interference effects for N450 amplitude (**Figure 2**). However, significantly shorter latency of the N450 to happy incongruent stimuli than happy congruent stimuli was found in MDDs. Previous studies have shown that the N450 was attenuated in folks with impaired ability to suppress competitive lexical information in incongruent trials (Mayes et al., 2005). Contrary to these findings, this study manifested that MDDs were characterized by significantly shorter N450 latency in response to happy incongruent stimuli than happy congruent stimuli. The finding may suggest that MDDs require early evaluation processes during incongruent stimuli with negative distractive word compared with congruent stimuli with positive distractive word, so as to perform the task at a normative level. Although the HCs failed to observe Stroop effect at N450 time window between congruent and incongruent stimuli during the Stroop task, a slice of examines have manifested that the effect is usually more robust when trials frequency is manipulated (Vanderhasselt and De, 2009).

With regard to P300, P300 amplitude of HC individuals was significantly greater when following sad incongruent stimuli relative to following sad congruent stimuli, and was maximal at central-parietal recording sites (**Figure 3**). The larger P300 amplitude indicates the stronger inhibition capability to incongruent stimulus, which is consistent with previous studies (Ramautar et al., 2006; Smith et al., 2010). Pearson’s correlations showed that a significant negative relationship between RT Stroop score and the difference in P300 amplitude for sad faces had emerged in the present study in HCs (**Supplementary Figure S2A**), which indicated that larger P300 amplitude difference between sad incongruent and sad congruent trials reflects greater inhibition capability and is corresponding to lower Stroop interference effect in RT. Unlike HCs, this pattern was absent in MDDs. The previous study has reported that patients with depression have diverse attention to emotional information relative to HCs, which is characterized by a higher degree of participation in emotional information (Liu et al., 2007). The finding may imply that the processing of depression on the sad face affects the handling of emotional conflicts of patients with MDD.

Thirdly, source localization analyses indicated that MDD subjects had significantly enhanced activity in rostral ACC (BA 24/32/33), PCC (BA 23/30/31), and insula (BA 13) within N450 time window in response to happy incongruent stimuli than happy congruent stimuli (**Figure 4A** and **Table 2**). Previous studies have shown that rostral ACC integrates emotions and conflicts (Kanske and Kotz, 2011), and are activated for conflict when stimuli are emotional (Egner et al., 2008). The current results indicated that response to conflict from negative distractive word was associated with activation in the rostral ACC. These results offer support for the longstanding view that the rostral ACC is involved in affective processing (Devinsky et al., 1995; Bush et al., 2000). Furthermore, substantiate previous data indicating that the rostral ACC is involved in processing task-irrelevant emotional stimuli (Bishop et al., 2004; Egner et al., 2008), and that its processing may be exclusive to the affective domain (Mohanty et al., 2007). Besides, similar work using fMRI on an emotional Stroop task manifested that severity of depressive symptoms was positively correlated with PCC activity in processing negative distractors (Kaiser et al., 2015), which was also considered indicative of evidence of impaired conflict processing that MDD patients processed increased internally directed attention to negative emotional stimuli. These findings suggested that negative emotional distraction leads to greater conflict in the high depressive symptoms of individuals and rostral ACC is involved in processing task-irrelevant emotional stimuli.

Finally, incongruent stimuli in HC subjects induced higher activity in IFG (BA 47) region at N450 time window compared with sad congruent stimuli (**Figure 4B** and **Table 2**). The defect of inhibition may undermine the adaptive emotional regulation strategy, this requires an individual to overcome and replace the initial negative interpretation of emotional induction. In fact, the deficiencies of inhibition have been confirmed in depression (Gotlib and Joormann, 2010). Saunders and Jentzsch (2014) found that participants with a high-depression symptom score processed difficulty with inhibition on an emotional-face Stroop task. In particular, a slice of studies have reported that the IFG is associated with successful cognitive function and executive function in the inhibition process (Whalen et al., 1998; Chiu et al., 2008; Morein-Zamir et al., 2014). Chiu et al. (2008) revealed enhanced activity in the region of right IFG to affective response inhibition. As Morein-Zamir et al. (2014) detected a significantly reduced activation when controlling for attentional processing, it indicated that low activation of the right IFC in ADHD was connected with the impaired response inhibition. HCs demonstrated increased right IFG activity to incongruent stimuli relative to congruent stimuli, which was suggesting of successful inhibition of emotional distraction. MDD patients lack this inhibitory effect on processing of emotional distraction. Furthermore, a significant effect of increased activity was found in region of the MFG for sad incongruent stimuli in HC group implies that attention may reorient to relevant targets from unrelated emotional distraction. This finding is in line with previous studies by Japee et al. (2015) which was suggestive of a crucial role of right MFG in reorienting of attention.

The statistical differences between groups for the sad incongruent task condition are illustrated in **Supplementary Figure S1**. The differences manifested an increase of activity in SPL and IPL (BA 7/40) within P300 time window (**Table 2**). Activation of the SPL can be caused when the subjects have to disengage their attention from fixation and move it to a cued location (Liu et al., 2003; Serences et al., 2004; Shomstein and Yantis, 2004). In the current work, the change in the SPL and IPL was interpreted as shift of attention from happy emotional distraction to location of sad facial expression in MDD individuals. We speculate that the higher activation in SPL in MDDs may reflect top-down attentional control.

From a clinical perspective, impairment of emotional conflict control is a major characteristic of MDD patients. The results of this study support the notion that depressive participants displayed dysfunction in the executive process and dysfunction of both emotional conflict monitoring and cognitive control processes. The major limitation of the present work is that relatively few electrodes are used in source localization. As emphasized in the previous study (Liu et al., 2017, 2018), high-density EEG can furnish both high spatial sampling density and large head coverage. Furthermore, there are many advanced tools for an effective network study (Yao et al., 2016; Hu et al., 2017), the introduction of which to them into major depression research would be meaningful. Finally, the research of functional and structural integrity of frontal pathways which are closely connected with emotional conflict among MDD patients should remain an important area for future study using high-density EEG.

## Conclusion

The present study investigated the differences in behavior and neural response to the emotional conflict in major depressive subjects and HCs, using a face–word Stroop task. Compared with HCs, abnormalities in MDDs in emotional conflict have been demonstrated through novel behavioral and neurophysiological evidence. As an example, MDD patients exhibited significant Stroop effects during the processing of happy incongruent and happy congruent trials, as well as the attenuated difference between P300 component to sad congruent stimuli and sad incongruent stimuli, relative to HCs. Source localization analyses indicated that MDDs had enhanced rostral ACC activity to happy incongruent stimuli than congruent stimuli, indicating that the rostral ACC is involved in processing task-irrelevant emotional stimuli. Furthermore, a higher activation in the region of right IFG was found in HCs for incongruent stimuli than for congruent stimuli. The increased activation in the right IFG was indicative of successful suppression of the emotional distraction in HCs, which was absent in MDD patients. These findings not only improve our understanding of the inhibition of MDD patients, but also pave the way for cognitive neuropsychological disorders modeling.

## Author Contributions

JL and XL conceived and designed the study. JZ, JL, and ZD acquired the data. JZ, JL, and XL analyzed and interpreted the data. All authors wrote the manuscript.

## Conflict of Interest Statement

The authors declare that the research was conducted in the absence of any commercial or financial relationships that could be construed as a potential conflict of interest.

## Footnotes

1 https://www.nimh.nih.gov/health/topics/depression/index.shtml

## Supplementary Material

FIGURE S1 Differences between groups. The statistical differences between MDDs and HCs for happy incongruent trials within N450 time window **(A)** and for sad incongruent trials within P300 time windows **(B)**, respectively. Yellow color indicates MDDs > HCs. Blue color indicates MDDs < HCs. The time periods for different components are shown in **Table 2**.

FIGURE S2 Relationship between difference in P300 amplitude and RT Stroop score. The difference in P300 amplitude between sad incongruent and sad congruent trials is significantly correlated with RT Stroop score for HCs **(A)**. However, it was not correlated with RT Stroop score for MDDs **(B)**. *X*-axis: RT Stroop score; *Y*-axis: Difference amplitude represents the difference in amplitude of the P300 on emotion-incongruent and emotion-congruent trials.
