# Supplementary figures and images for: Neural Basis of the Emotional Conflict Processing in Major Depression: ERPs and Source Localization Analysis on the N450 and P300 Components
Source: Front Hum Neurosci. 2018 May 29;12:214. doi: 10.3389/fnhum.2018.00214 (PMC5986884; doi:10.3389/fnhum.2018.00214)

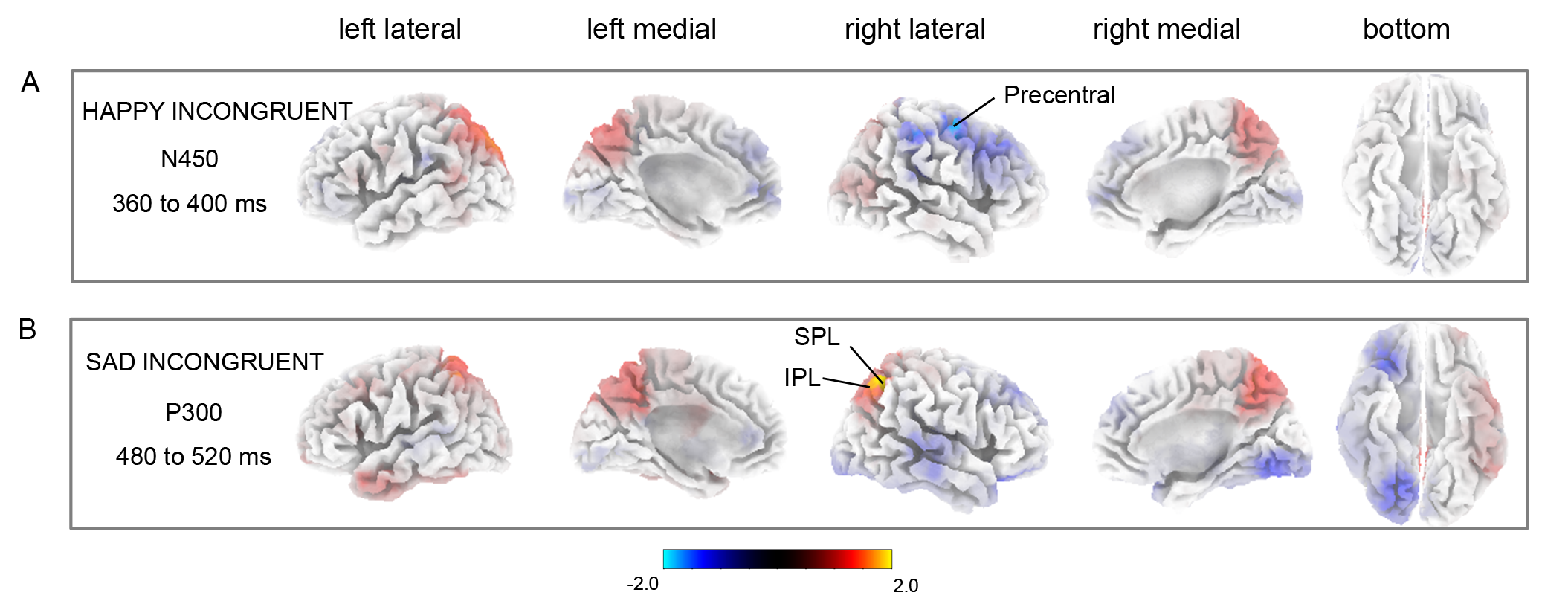

Supplement: FIGURE S1 — Differences between groups. The statistical differences between MDDs and HCs for happy incongruent trials within N450 time window (A) and for sad incongruent trials within P300 time windows (B), respectively. Yellow color indicates MDDs > HCs. Blue color indicates MDDs < HCs. The time periods for different components are shown in Table 2. [file Image_1.TIF]

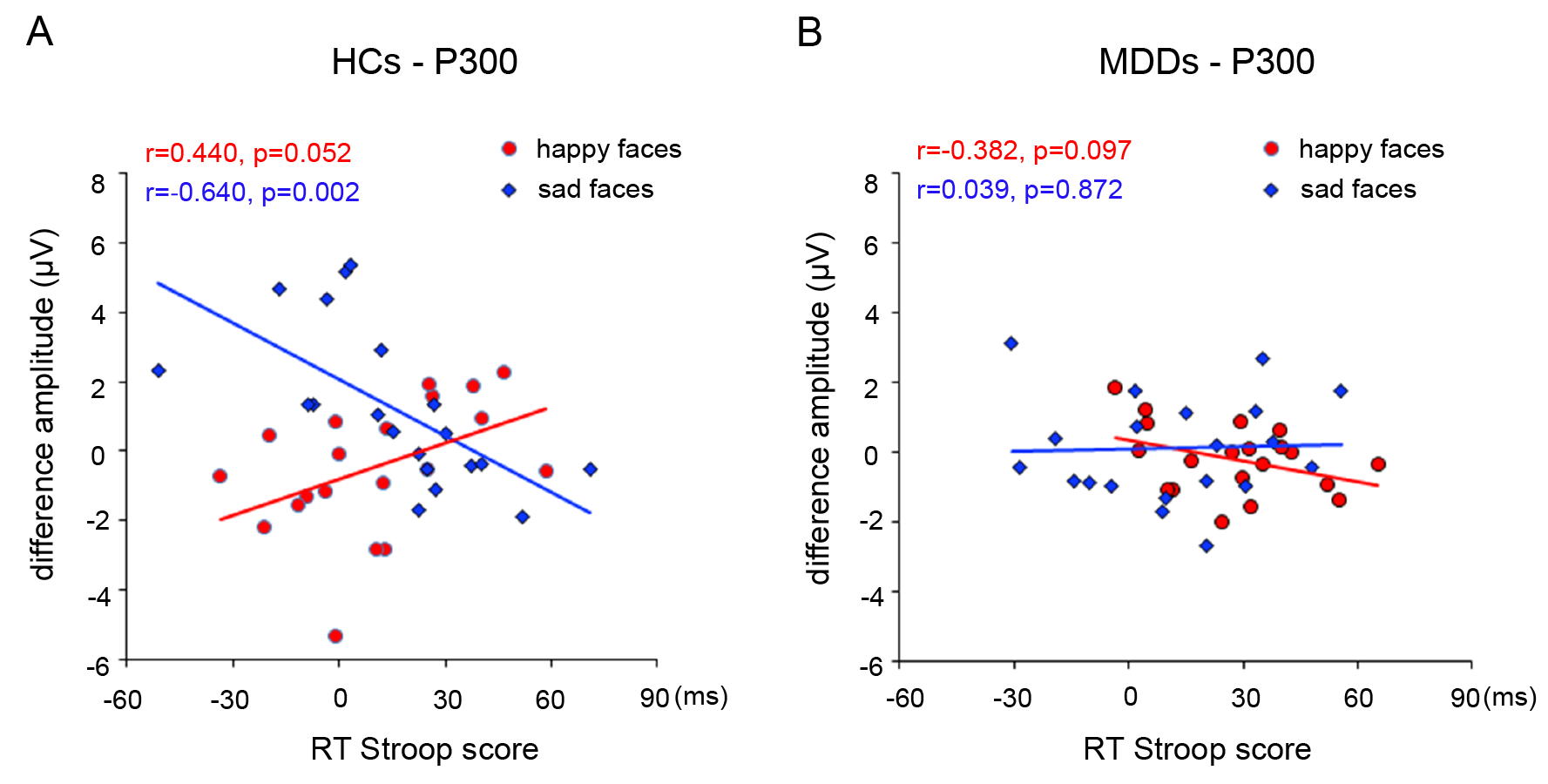

Supplement: FIGURE S2 — Relationship between difference in P300 amplitude and RT Stroop score. The difference in P300 amplitude between sad incongruent and sad congruent trials is significantly correlated with RT Stroop score for HCs (A). However, it was not correlated with RT Stroop score for MDDs (B). X-axis: RT Stroop score; Y-axis: Difference amplitude represents the difference in amplitude of the P300 on emotion-incongruent and emotion-congruent trials. [file Image_2.TIF]
